# Spatio-temporal profile, phenotypic diversity, and fate of recruited monocytes into the post-ischemic brain

**DOI:** 10.1186/s12974-016-0750-0

**Published:** 2016-11-04

**Authors:** Lidia Garcia-Bonilla, Giuseppe Faraco, Jamie Moore, Michelle Murphy, Gianfranco Racchumi, Jayashree Srinivasan, David Brea, Costantino Iadecola, Josef Anrather

**Affiliations:** Feil Family Brain and Mind Research Institute, Weill Cornell Medicine, 407 East 61st Street RR409, New York, NY 10065 USA

**Keywords:** Monocytes, Macrophages, Cerebral ischemia, CCR2, CX3CR1, Ly6C

## Abstract

**Background:**

A key feature of the inflammatory response after cerebral ischemia is the brain infiltration of blood monocytes. There are two main monocyte subsets in the mouse blood: CCR2^+^Ly6C^hi^ “inflammatory” monocytes involved in acute inflammation, and CX3CR1^+^Ly6C^lo^ “patrolling” monocytes, which may play a role in repair processes. We hypothesized that CCR2^+^Ly6C^hi^ inflammatory monocytes are recruited in the early phase after ischemia and transdifferentiate into CX3CR1^+^Ly6C^lo^ “repair” macrophages in the brain.

**Methods:**

CX3CR1^GFP/+^CCR2^RFP/+^ bone marrow (BM) chimeric mice underwent transient middle cerebral artery occlusion (MCAo). Mice were sacrificed from 1 to 28 days later to phenotype and map subsets of infiltrating monocytes/macrophages (Mo/MΦ) in the brain over time. Flow cytometry analysis 3 and 14 days after MCAo in CCR2^−/−^ mice, which exhibit deficient monocyte recruitment after inflammation, and NR4A1^−/−^ BM chimeric mice, which lack circulating CX3CR1^+^Ly6C^lo^ monocytes, was also performed.

**Results:**

Brain mapping of CX3CR1^GFP/+^ and CCR2^RFP/+^ cells 3 days after MCAo showed absence of CX3CR1^GFP/+^ Mo/MΦ but accumulation of CCR2^RFP/+^ Mo/MΦ throughout the ischemic territory. On the other hand, CX3CR1^+^ cells accumulated 14 days after MCAo at the border of the infarct core where CCR2^RFP/+^ accrued. Whereas the amoeboid morphology of CCR2^RFP/+^ Mo/MΦ remained unchanged over time, CX3CR1^GFP/+^ cells exhibited three distinct phenotypes: amoeboid cells with retracted processes, ramified cells, and perivascular elongated cells. CX3CR1^GFP/+^ cells were positive for the Mo/MΦ marker Iba1 and phenotypically distinct from endothelial cells, smooth muscle cells, pericytes, neurons, astrocytes, or oligodendrocytes. Because accumulation of CX3CR1^+^Ly6C^lo^ Mo/MΦ was absent in the brains of CCR2 deficient mice, which exhibit deficiency in CCR2^+^Ly6C^hi^ Mo/MΦ recruitment, but not in NR4A1^−/−^ chimeric mice, which lack of circulating CX3CR1^+^Ly6C^lo^ monocytes, our data suggest a local transition of CCR2^+^Ly6C^hi^ Mo/MΦ into CX3CR1^+^Ly6C^lo^ Mo/MΦ phenotype.

**Conclusions:**

CX3CR1^+^Ly6C^lo^ arise in the brain parenchyma from CCR2^+^Ly6C^hi^ Mo/MΦ rather than being de novo recruited from the blood. These findings provide new insights into the trafficking and phenotypic diversity of monocyte subtypes in the post-ischemic brain.

**Electronic supplementary material:**

The online version of this article (doi:10.1186/s12974-016-0750-0) contains supplementary material, which is available to authorized users.

## Background

Activation of brain-resident cells and mobilization of circulating leukocytes is a key feature of the inflammatory response after cerebral ischemia [1]. In animal models as in human stroke, post-ischemic brain inflammation is characterized by infiltration of hematogenous immune cells, monocyte-derived tissue macrophages (Mo/MΦ) being one of the predominant cell types [2–5]. Growing evidence suggests that Mo/MΦ invading the ischemic brain can contribute to both brain injury [6, 7] and repair [8, 9]. These opposing roles reflect the heterogeneity of the monocyte-macrophage lineage, which may adopt diverse phenotypes depending on environmental cues [10].

Two predominant subsets of circulating monocytes (CD115+ leukocytes) are found in the blood of rodents and humans: “inflammatory ” and “patrolling” monocytes. In mice, inflammatory monocytes are characterized by the expression of the C-C chemokine receptor type 2 (CCR2), a critical molecule involved in the rapid recruitment of the cells into inflamed tissues [11]. They also express high levels of lymphocyte antigen 6 complex locus C1 (Ly6C) but low to intermediate levels of C-X(3)-C motif chemokine receptor 1 (CX3CR1). On the other hand, CX3CR1, the high-affinity functional chemokine receptor for fractalkine (CX3CL1), is enriched in patrolling monocytes, which do not express Ly6C and CCR2 or express them at low levels. Patrolling monocytes (Ly6C^lo^CCR2^−^CX3CR1^+^) have longer half-life and crawl on the vessel luminal wall orchestrating the disposal of damaged endothelial cells [12]. In addition, patrolling monocytes can rapidly invade tissues in response to tissue damage or infection [13, 14]. Patrolling monocytes were previously thought to originate from circulating inflammatory CD115^+^Ly6C^hi^ monocytes [14, 15], but recent data suggest that they originate from distinct myeloid progenitors by the activity of the nuclear receptor subfamily 4, group A, member 1 transcription factor NR4A1 (Nur77) in the BM [16]. Furthermore, depletion of CD115^+^Ly6C^hi^ monocytes in the circulation does not affect the numbers of CD115^+^Ly6C^lo^ monocytes, reducing the likelihood that the sole precursors of “patrolling” monocytes are CD115^+^Ly6C^hi^ cells [17].

Little is known about the monocytes subtypes invading the ischemic brain, their morphology, and their fate once in the tissue. Monocytes infiltrating the ischemic brain acquire morphological and functional properties of tissue macrophages and can persist in the brain parenchyma for extended periods of time [7, 8, 18–20]. However, the trafficking of inflammatory and patrolling monocytes into the ischemic brain, their relationship to the development of tissue injury, and their long-term fate remain unknown.

Here, we investigated the temporal profile, spatial distribution, and phenotype of inflammatory (CCR2^+^Ly6C^hi^) and patrolling (CX3CR1^+^Ly6C^lo^) monocyte subpopulations in transient focal cerebral ischemia. Using a mouse model to track CCR2 and CX3CR1 expression in hematogenous cells, we found that CCR2^+^ Mo/MΦ invade the brain in the acute phase and give rise to CX3CR1^+^ Mo/MΦ during the subacute and chronic phase of the injury, without the participation of circulating CX3CR1^+^ Mo. The findings identify CCR2^+^Ly6C^hi^ monocytes as the primary monocytic cell infiltrating the ischemic brain, where they undergo phenotypic transformation during the evolution of the damage. Such phenotypic diversity may underlie the multiplicity of roles attributed to Mo/MΦ in ischemic brain injury.

## Methods

### Mice

Experiments were performed in 8–12-week-old male CCR2^RFP/+^CX3CR1^GFP/+^, CCR2^−/−^, or NR4A1^−/−^ on a C57Bl/6J genetic background. Age-matched wild-type (WT) mice (C57Bl/6J, Jackson Laboratory, Bar Harbor, ME) served as controls. All animal experiments were performed in accordance with the ARRIVE guidelines [21].

### Middle cerebral artery occlusion

Transient focal cerebral ischemia was induced using the intraluminal filament model of middle cerebral artery occlusion (MCAo), as described previously [22]. Under isoflurane anesthesia (maintenance 1.5-2 %), a heat-blunted nylon suture (6/0) was inserted into the right external carotid artery of anesthetized mice and advanced until it obstructed the middle cerebral artery (MCA). This was confirmed by cerebral blood flow (CBF) measured using transcranial laser Doppler flowmetry (Periflux System 5010, Perimed, King Park, NY) in the territory of the right MCA (2 mm posterior, 5 mm lateral to bregma). The filament was left in place for 35 min and then withdrawn. Only animals that exhibited a reduction in CBF of >85 % during MCA occlusion and in which CBF recovered by >80 % after 10 min of reperfusion were included in the study. Inadequate ischemia-reperfusion was found to be <4 % in the study and not different among groups. Rectal temperature was monitored and kept constant (37.0 ± 0.5 °C) during the surgical procedure and in the recovery period until the animals regained full consciousness. No differences were found in mortality rate (<1 %) among groups.

### Bone marrow CCR2^RFP/+^CX3CR1^GFP/+^ and NR4A1^−/−^ chimera generation

CCR2^RFP/RFP^ (JAX 017586) and CX3CR1^GFP/GFP^ (JAX 005582) mice were crossed to obtain heterozygous mice that underwent chromosome recombination between the CCR2 and CX3CR1 loci (CCR2^RFP/+^CX3CR1^GFP/+^ mice). Recombination of CCR2 and CX3CR1 loci was confirmed by polymerase chain reaction (PCR) using tail DNA. DNA was purified from tails using NaOH extraction [23]. Target primer sequences were CX3CR1_for 5′-TCTGCTGAGGCCTGTTATTTG-3′ and CX3CR1_rev 5′-ACCAGACCGAACGTGAAGAC-3′; enhanced green fluorescent protein (EGFP)_for 5′-ctgaccctgaagttcatctgc-3′ and EGFP_rev 5′-gcttgtcggccatgatataga-3′; CCR2_for 5′-GGATTAAGGAATTTGGCATTTG-3′, CCR2_rev 5′-GGAGTAGAGTGGAGGCAGGA-3′, and red fluorescent protein (RFP)_rev 5′-CTTGATGACGTCCTCGGAG-3′. All primers were purchased from Invitrogen Life Technologies (Grand Island, NY). qRT-PCR was conducted with 3 μl of diluted DNA (1:10 dilution), in duplicate 15 μl reactions using the Maxima SYBR Green/ROX qPCR Master Mix (2×) (Thermo Scientific, Pittsburgh, PA). The reactions were incubated at 50 °C for 2 min and then at 95 °C for 10 min. A polymerase chain reaction cycling protocol consisting of 15 s at 95 °C and 1 min at 60 °C for 45 cycles was employed. Genotyping results were based on melting curve analysis. Whole-body irradiation was performed in 7-week-old male WT mice with a lethal dose of 9.5 Gy of γ radiation using a ^137^Cs source (Nordion Gammacell® 40 Exactor, Theratronics, Ottawa, Canada). Eighteen hours later, irradiated mice were transplanted with BM cells (2 × 10^6^, i.v.) isolated from 7-week-old donor CCR2^RFP/+^CX3CR1^GFP/+^ mice. Donor BM cells were flushed out with 2–3 ml of phosphate-buffered saline (PBS) from the femur and tibias, filtered through a 40-μm nylon mesh and counted for viability before injection. Transplanted mice were housed in cages with sulfatrim pellets for the first 2 weeks after irradiation. The restricted expression of green fluorescent protein (GFP) in hematogenous CX3CR1 cells allows for differentiation of infiltrating CX3CR1^GFP/+^ monocytes from central nervous system (CNS)-resident CX3CR1^+^ microglia. In a distinct group of WT mice, irradiation with head protection was performed using a custom designed lead shield (2.5 cm thickness) under the aforementioned irradiation procedure. For NR4A1^−/−^ chimera generation, WT mice were lethally irradiated as described above, and donor BM from NR4A1^−/−^ mice (JAX 006187) was used to produce WT mice with NR4A1-null leukocytes. Deletion of NR4A1 in blood leukocytes was analyzed in the chimeric mice by determining the absence of Ly6C^lo^ monocytes in blood by flow cytometry.

### Image analysis and cell counting of infiltrating CX3CR1^GFP/+^ and CCR2^RFP/+^ cells

**Fig. 1:**
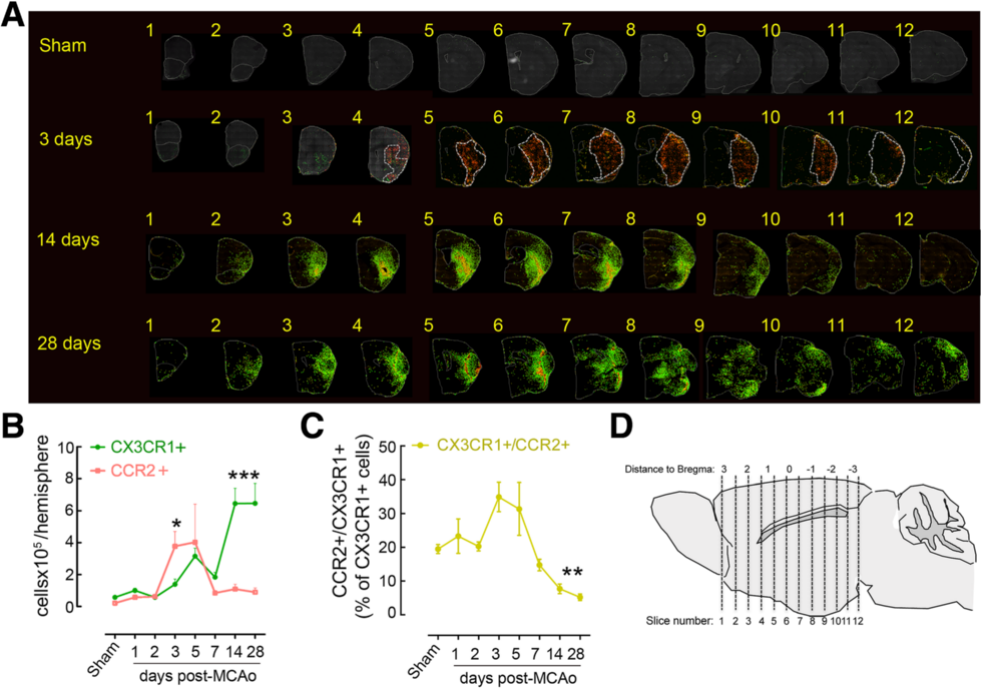
Spatio-temporal profile of CX3CR1^GFP/+^ and CCR2^RFP/+^ Mo/MΦ infiltration after stroke. **a** Anatomical localization of CCR2-RFP (*red*) and CX3CR1-GFP (*green*) cells infiltrating the ischemic hemisphere in coronal brain sections of CCR2^RFP/+^CX3CR1^GFP/+^chimeric mice 3, 14, or 28 days after MCAo and in sham mice. Infarct volume was outlined in serial cresyl violet stained sections 3 days after MCAo and overlaid on fluorescent images (dotted line). **b** Quantification of infiltrating CX3CR1^GFP/+^ and CCR2^RFP/+^ cells in sham mice and stroke mice 1, 2, 3, 5, 7, 14, and 28 days after MCAo (*n* = 3–4 mice/group). *p < 0.05 and ***p < 0.001 vs. Sham. **c** Quantification of infiltrating CX3CR1^GFP/+^CCR2^RFP/+^ double-positive cells in sham mice and stroke mice 1, 2, 3, 5, 7, 14, and 28 days after MCAo (*n* = 3–4 mice/group). **p < 0.01 vs. Sham. **d** Diagram depicting the brain-sectioning method. The brain was sectioned in 12 coronal slices at 600-μm intervals, beginning from the frontal pole and proceeding in caudal direction

Mice were perfused transcardially with ice-cold PBS followed by 4 % paraformaldehyde in PBS. Brains were removed, stored overnight in the same fixative at 4 °C, and then submerged in 30 % sucrose solution for 2 days and frozen. Using a cryostat, 12 serial coronal sections (thickness 16 μm) were cut every 600 μm from the frontal cortex to the caudal hippocampus (+3.14 to −3.46 bregma) and then mounted on slides (Fig. 1b). Fluorescent images were acquired by a scientific monochrome CCD camera (Retiga EXi, QImaging) using fluorescein isothiocyanate (FITC) (CX3CR1-GFP) and Texas Red (CCR2-RFP) filter sets through a 10× objective on a Nikon TE2000 microscope equipped with a motorized stage. Acquisition was controlled via software (IPLab, Biovision Technologies, Exton, PA) using a custom script. Individual image files (80 for each hemisphere) were combined using the ImageJ software (National Institute of Health, version 1.45) by invoking a stitching algorithm [24]. A custom ImageJ macro was used to semi-automatically count cells identified after thresholding the image based on pixel values. In lesions at 14 and 28 days after ischemia, we observed a densely populated lesion core where individual cells could not be unequivocally separated from neighboring cells. Therefore, these areas were excluded from the analysis. For presentation purposes, binary image masks of both green and red channel images generated for cell counting were superimposed on a grayscale image obtained from the FITC channel image by running the ImageJ “Remove Outliers” command once and the ImageJ “Smooth” command twice.

### Immunofluorescence

Frozen brain sections were permeabilized with 0.5 % Triton X-100 (Sigma) in PBS (PBST) for 30 min at room temperature, blocked with 5 % normal donkey serum (NDS) in 0.1 % in PBST and incubated with antibodies against the neuronal marker NeuN (1: 100, mouse monoclonal; Millipore), the endothelial cell marker glucose transporter 1 (GLUT-1) (1:200, rabbit polyclonal antibody; Calbiochem), the astrocytic marker glial fibrillary acidic protein (GFAP) (1:200, mouse monoclocal; Sigma-Aldrich), the microglia/macrophage marker ionized calcium binding adaptor molecule 1 (Iba1) (1:500, rabbit polyclonal antibody; Wako Chemicals), the pericyte marker beta-type platelet-derived growth factor receptor (PDGFRβ) (1:200, rat polyclonal antibody; Abcam), the vascular smooth muscle cells marker α-actin (1:800, rabbit polyclonal antibody; Abcam), or the oligodendrocyte marker Olig2 (1:200, rabbit polyclonal antibody; Abcam) in 1% NDS-0.1 % PBST at 4 °C overnight. Ki67 was used as a cell proliferation marker (1: 200, rabbit polyclonal; Millipore). After three 10-min washes with 0.1 % PBST, brain slices were incubated with a FITC-, DyLight 405-, or a Cy5-conjugated secondary antibody (1:200; Jackson ImmunoResearch Laboratories). For antigen retrieval, boiling 10 mM sodium citrate buffer was applied on brain sections for 5 min before initiating immunostaining for NeuN or Ki67. Brain sections were then mounted on slides and immunostaining was visualized by confocal microscopy (Leica TCS SP5, Mannheim, Germany) or with an epifluorescence microscope (IX83 Inverted Microscope, Olympus, Center Valley, PA). The specificity of the immunofluorescence was verified in pilot studies by omission of the primary antibody.

The proportion of morphological phenotypes of CX3CR1^GFP/+^ cells was quantified within the ischemic hemisphere in images acquired with a 40× objective from 1.3, 0.7, −0.1, −0.5, and −1.1 bregma coronal brain sections at 14 days post-MCAO that were stained for GLUT-1 using ImageJ software. For the analysis of Ki67^+^ cells, coronal sections were scanned with a confocal microscope and Ki67^+^ and CX3CR1^GFP/+^ cells were quantified from tile-scanned images using ImageJ software.

### Brain mononuclear cell isolation

Mice were transcardially perfused under deep anesthesia with 30 ml of heparinized saline to remove blood cells. The ischemic hemisphere was separated from the cerebellum and olfactory bulb and homogenized with a dounce homogenizer in RPMI Medium 1640 with Phenol Red. Then, the cell suspension was subjected to discontinuous 70 %/25 % Percoll (GE Healthcare, Piscataway, NJ) density gradient centrifugation (30 min, 500 g, 4 °C). Enriched-mononuclear cells were collected from the interphase, washed with Hepes-HBSS buffer (138 mM NaCl, 5 mM KCl, 0.4 mM Na_2_HPO_4_, 0.4 mM KH_2_PO_4_, 5 mM d-Glucose, 10 mM Hepes, pH 7.4), and resuspended in 2 % fetal bovine serum (FBS) in PBS FACS buffer for staining and FACS analysis.

### Flow cytometry and cell sorting

Rat monoclonal antibodies used for flow cytometry analysis include CD45-BV 510 or CD45-APC (clone 30 F-11), Ly6C-FITC (clone HK1.4), CX3CR1-PE (clone SA011F11), Ly6G-PerCP-Cy5.5 (clone 1A8), CD8a-PerCP-Cy5.5 (clone 53–6.7), CD115-PE (clone AFS98), NK1.1-APC (clone PK136), and CD11b-APC-Cy7 (clone M1/70) from BioLegend (San Diego, California) and CCR2-APC (clone 475301) from R&D. For flow cytometric analysis of blood leukocytes, 100 μl of blood was withdrawn by cardiac puncture from mice under deep anesthesia immediately before sacrifice. Erythrocytes were lysed by incubating the blood three times with 5 ml erythrocyte lysis buffer (0.15 M NH_4_Cl, 1 mM KH_2_CO_3_, and 0.1 mM Na-EDTA) for 5 min at room temperature. Blood leukocytes were resuspended in FACS buffer. Cells were blocked with CD16/CD32 antibody for 10 min at 4 °C, stained with predetermined optimal concentrations of antibodies for 20 min at 4 °C, and analyzed on a MACSQuant (Miltenyi Biotec) or a FACSCalibur (Becton-Dickinson) cytometer. Dead cells and debris were excluded based on side and forward scatter profiles (SSC and FSC, respectively). Appropriate isotype control antibodies, “fluorescence minus one” staining and staining of negative populations were used to establish compensation parameters and sorting gates. Absolute cell numbers and frequencies were recorded. Data were analyzed using FlowJo software (FlowJo, Ashland, OR).

### Data analysis

Data are expressed as mean ± SE. Intergroup differences were analyzed using a Student unpaired *t* test or one-way ANOVA, as appropriate. Differences were considered statistically significant for *p* < 0.05. Animals were randomly assigned to treatment and control groups and analysis was performed in a blinded fashion.

## Results

### CCR2^+^ Mo/MΦ accumulate in the ischemic brain before CX3CR1^+^ Mo/MΦ

To investigate brain trafficking of monocytes after cerebral ischemia, we generated CX3CR1^GFP/+^CCR2^RFP/+^ BM chimeric mice. GFP and RFP expression enables to simultaneously track CX3CR1^+^Ly6C^lo^ and CCR2^+^Ly6C^hi^ monocyte recruitment, while the generation of BM chimeras, allows for differentiation between infiltrating CX3CR1^GFP/+^ monocytes and CNS-resident (CX3CR1^+^) microglia [25]. Histological analysis of brains of CX3CR1^GFP/+^CCR2^RFP/+^ chimeras that underwent MCAo revealed a significant infiltration of monocytes in the ischemic brain (Fig. 1). CCR2^RFP/+^ cells rapidly increased in the ischemic hemisphere 3 days after MCAo, evenly infiltrating the injured tissue, identified by Nissl stain of adjacent brain sections, with minimal penetration of the healthy tissue (Fig. 1a). During the subacute and chronic phase of injury (day 14–28 after MCAo), CCR2^RFP/+^ cells were confined to the lesion core and their number decreased elsewhere (Fig. 1a). The number of CCR2^RFP/+^ cells was highly dense in the lesion core 14–28 days after ischemia, which made it impossible to differentiate single cells, and therefore, this region was excluded for the quantitative analysis (see the “Methods” section; Fig. 1b). CX3CR1^GFP/+^ cell numbers increased moderately in the first 7 days after MCAo and peaked at 14–28 days post-ischemia (Fig. 1a-b). CX3CR1^GFP/+^ cells delimited the CCR2^RFP/+^ cell-laden core at 14 days after MCAo and were also found, albeit sparsely, throughout the entire lesion. This anatomical distribution remained unmodified 28 days after MCAo (Fig. 1a). In brains of mice undergoing sham surgery, CCR2^RFP/+^ and CX3CR1^GFP/+^ cells were scarce and localized mostly in the choroid plexus, olfactory bulbs and meninges (Fig. 1a). Moreover, the number of CCR2^RFP/+^CX3CR1^GFP/+^ double-positive cells was homogenous among the sham and 1–2 days post-ischemia groups (20 ± 3 % CCR2^RFP/+^CX3CR1^GFP/+^ of total CX3CR1^GFP/+^) and increased 3–5 days after MCAo (33 ± 3 %) before significantly decreasing between 7 and 28 days after MCAo (6 ± 2 %, Fig. 1c).

Because irradiation of the head may induce an inflammatory response [26] that may alter immune cell infiltration, CX3CR1^GFP/+^CCR2^RFP/+^ chimeric mice were also obtained by irradiation with head shielding (Additional file 1: Figure S1). However, head shielding had no effect on brain distribution of CCR2^RFP/+^ and CX3CR1^GFP/+^ cells 3 and 14 days after stroke, when compared to chimeric mice without head protection (Additional file 1: Figure S1A–B).

### Infiltrating CX3CR1^+^ cells but not CCR2^+^ cells displayed different morphological phenotypes

**Fig. 2:**
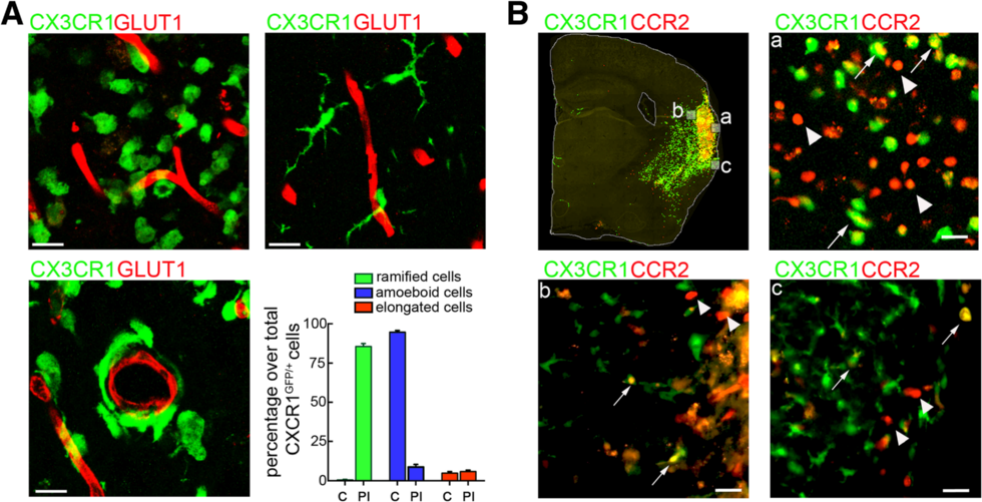
Morphological diversity of CX3CR1^GFP/+^ and CCR2^RFP/+^ infiltrating Mo/MΦ. **a** CX3CR1^GFP/+^ cells (*green*) show three different phenotypes at 14 days after MCAo: amoeboid cells (upper left), ramified cells (upper right), and elongated cells located along the vessels (*lower left*). Blood vessels were immunostained with the endothelial marker GLUT1 (red). *Scale bars* represent 15 μm. *Graph bars* represent the frequencies for each described phenotype in the core (*C*) and periphery (peri-infarct; *PI*) of the lesion (*n* = 4 mice/group). **b** CCR2^RFP/+^ cells (*red*), which are localized in the core of the ischemic lesion (*a*), have a round shape (arrowheads), while CX3CR1^GFP/+^CCR2^RFP/+^ double-positive cells (*arrows*), have an amoeboid or spindle shape with lower level of RFP expression than CCR2^RFP/+^ cells (*a* and *b*). CX3CR1^GFP/+^ cells acquire a ramified phenotype as they localized in the periphery (*b* and *c*). *Scale bars* represent 20 μm

CX3CR1^GFP/+^ cells exhibited three distinct phenotypes 14 and 28 days after MCAo (Fig. 2a): (i) cells with an amoeboid morphology and no plasmalemmal processes, classified as *amoeboid cells* (left panel); (ii) cells with arborized processes, named *ramified cells* (central panel); and (iii) cells with elongated shape located along the vessels termed *perivascular cells* (right panel). Amoeboid CX3CR1^GFP/+^ cells were often localized in the ischemic core and adjacent areas (95 ± 1.2 % of the total CX3CR1^GFP/+^ cells), which were mainly populated by CCR2^+^ cells. Ramified CX3CR1^GFP/+^ cells were predominant at the periphery of the lesion (85 ± 1.9 % of the total CX3CR1^GFP/+^ cells) and were not seen in the core at 14 days after MCAo. Perivascular CX3CR1^GFP/+^ cells were closely associated with blood vessels identified by the endothelial marker GLUT1 and were observed in both core (5 ± 1.0 %) and peri-infarct (5.8 ± 1.0 % of the total CX3CR1^GFP/+^ cells) regions (Fig. 2a). These perivascular cells exhibited elongated profiles along the major axis of the vessel or encircled the entire vessels’ circumference in vascular cross sections. Similar distributions were observed 28 days after MCAo (data not shown). Head shielding did not affect the phenotype distribution of CX3CR1^GFP/+^ cells (Additional file 1: Figure 1D). In contrast, infiltrating CCR2^RFP/+^ cells showed a uniform round shape without apparent processes, distinctive of amoeboid macrophages, and as described above, their distribution was limited to the ischemic core 14 days after MCAo (Fig. 2b). Some CCR2^RFP/+^CX3CR1^GFP/+^ double-positive cells showed cellular processes (Fig. 2B), suggesting that CCR2^+^ cells may acquire a CX3CR1 phenotype in the tissue.

**Fig. 3:**
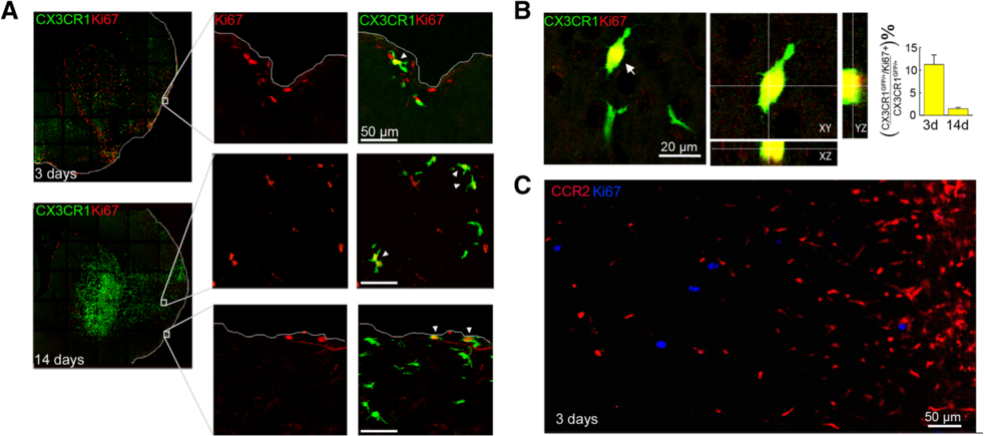
CX3CR1^GFP/+^ Mo/MΦ proliferate in the ischemic brain. **a** Co-staining of CX3CR1^GFP/+^ with the proliferation marker Ki67 shows that some CX3CR1^GFP/+^ Mo/MΦ (*green*) are Ki67 positive (*red*) 3 and 14 days after MCAo. Scale bars represent 50 μm. **b** Orthogonal views of CX3CR1^GFP/+^ (*green*) and Ki67+ (*red*) showing cellular co-localization. On the *right*, percentage of double-positive CX3CR1^GFP/+^/Ki67+ cells over the total CX3CR1^GFP/+^ cells in the ischemic brain 3 and 14 days after MCAo (*n* = 4 mice/group). **c** Ki67+ cells do not co-localized with CCR2^RFP/+^ cells in the ischemic region 3 days after MCAo

To investigate whether proliferation of brain recruited Mo/MΦ may also contribute to the increase in the number of accumulated Mo/MΦ over the time after stroke, we performed immunohistochemistry with the proliferation marker Ki67 (Fig. 3). Some CX3CR1^GFP/+^ macrophages on the brain surface were positive for Ki67 (11.3 ± 2.1 % of total CX3CR1^GFP/+^ cells) 3 days after MCAo (Fig. 3a) and 14 days after MCAo, Ki67 co-labeled with CX3CR1^GFP/+^ Mo/MΦ macrophages localized either in the parenchyma or on the brain surface (Fig. 3a). However, at this time point, the number of CX3CR1^GFP/+^Ki67^+^ Mo/MΦ accounted for only 1.0 ± 0.3 % of total CX3CR1^GFP/+^ cells (Fig. 3b). We did not observe CCR2^RFP/+^Ki67^+^ cells (Fig. 3c). Ki67 staining in head-shielded mice also revealed proliferation of CX3CR1^GFP/+^ Mo/MΦ (Additional file 1: Figure S1C).

### CX3CR1^GFP/+^ cells are phenotypically distinct from endothelial cells, smooth muscle cells, pericytes, oligodendrocytes, neurons, and astroglia

**Fig. 4:**
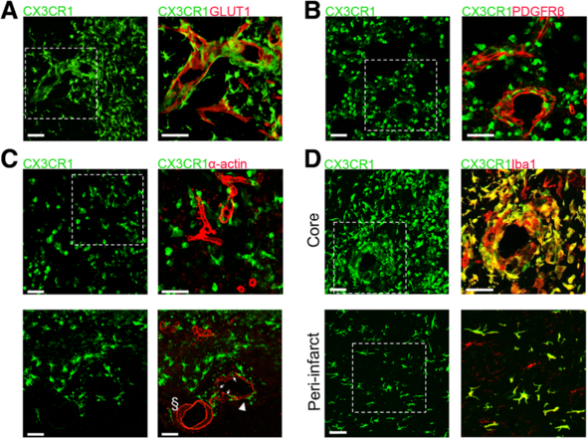
CX3CR1^GFP/+^ Mo/MΦ do not express vascular markers. CX3CR1^GFP/+^ cells do not express endothelial (GLUT1) (**a**), pericyte (PDGFRβ) (**b**), or smooth muscle cells (α-actin) (**c**) markers 14 days after MCAo but are positive for the microglia/macrophage marker Iba1 (**d**). *Scale bars* represent 50 μm. **c** Disorganized blood vessel (*arrowhead*) shows spotty α-actin staining and surrounding CX3CR1^GFP/+^ cells (*thin arrows*), as compared with an adjacent intact vessel (*§*), which shows uniform α-actin staining and no associated CX3CR1^GFP/+^ cells

Next, we sought to determine if CX3CR1^GFP/+^ cells differentiated into other cells types (Fig. 4). CX3CR1 is expressed by BM-derived endothelial progenitor cells [27], and BM-derived CX3CR1^+^ progenitors can contribute to neointimal smooth muscle cell formation [28, 29]. In addition, pericyte progenitors reside in the BM compartment from where they are mobilized after stroke [30, 31]. Therefore, considering their vascular proximity, we determined whether CX3CR1^GFP/+^ cells expressed endothelial, pericyte or vascular smooth muscle cell markers. However, GLUT1^+^ endothelial cells, PDGFRβ^+^ pericytes, and α-actin^+^ smooth muscle cells, while closely associated with CX3CR1^GFP/+^ cells, did not express GFP (Fig. 4a–c). In contrast, amoeboid, ramified, and perivascular CX3CR1^GFP/+^ cells were all positive for the microglia/MΦ marker Iba1 (Fig. 4d; Additional file 2: Figure S2), indicating their myeloid origin. CX3CR1^GFP/+^ Mo/MΦ did not show expression of astrocytic (GFAP), oligodendrocyte (Olig2), or neuronal (NeuN) markers (Additional file 3: Figure S3).

### Infiltration of CCR2^+^Ly6C^hi^ and CCR2^−^Ly6C^lo^ cells in CCR2- or NR4A1-null mice after ischemic injury

To investigate whether the late surge of CX3CR1^+^ Mo/MΦ was due to active recruitment of circulating CX3CR1^+^Ly6C^lo^ monocytes or to transdifferentiation of invading CCR2^+^Ly6C^hi^ into CX3CR1^+^Ly6C^lo^ Mo/MΦ, we studied Mo/MΦ dynamics in CCR2^−/−^ mice, in which tissue recruitment of CCR2^+^Ly6C^hi^ monocytes is impaired [32] and in chimeric mice lacking NR4A1^−/−^, a transcription factor required for the generation of CX3CR1^+^Ly6C^lo^ circulating monocytes [16]. These studies were conducted by flow cytometry using Ly6C expression as a surrogate marker for CCR2^+^ and CX3CR1^+^ Mo/MΦ identification. To validate this approach, we analyzed the blood of CX3CR1^GFP/+^CCR2^RFP/+^ chimeric mice by flow cytometry. This analysis identified that GFP^hi^RFP^lo^ cells corresponded to Ly6C^lo^ monocytes (CD45^+^Ly6G^−^CD11b^+^Ly6C^lo^ cells), whereas GFP^int^RFP^hi^ cells corresponded to Ly6C^hi^ monocytes (CD45^+^Ly6G^−^CD11b^+^Ly6C^hi^ cells) (Additional file 4: Figure S4). NK cells and some CD8 cells also expressed RFP, as previously reported [25], but neutrophils did not express either RFP or GFP.

**Fig. 5:**
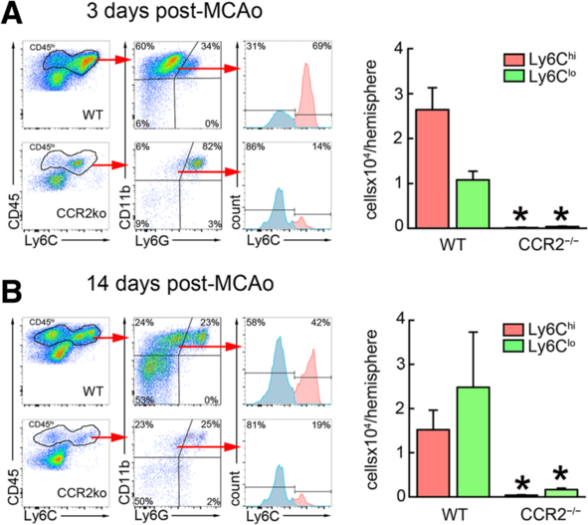
Ly6C^hi^ and Ly6C^lo^ Mo/MΦ do not accumulate in the brain of CCR2^−/−^ mice after MCAo. **a** Flow cytometry analysis of infiltrating Mo/MΦ, identified by high expression of CD45 and CD11b and low expression of Ly6G (CD45^hi^CD11b^+^Ly6G^−^), shows an increase of Ly6C^hi^ Mo/MΦ as compared with Ly6C^lo^ Mo/MΦ in the wild type (WT) mouse 3 days after MCAo. The accumulation of both Ly6C^hi^ and Ly6C^lo^ Mo/MΦ is abolished in CCR2^−/−^ mice. **b** Brain-resident Ly6C^lo^ Mo/MΦ outnumbered Ly6C^hi^ Mo/MΦ in WT mice 14 days after MCAo, while neither Ly6C^hi^ or Ly6C^lo^ Mo/MΦ increased in CCR2^−/−^ mice (*n* = 4 mice/group). *p < 0.05 vs. WT

In agreement with previous studies, flow cytometry of blood from CCR2^−/−^ mice showed reduced number of Ly6C^hi^ cells, because CCR2 is required for egression of Ly6C^hi^ monocytes from BM [33]. However, the number of circulating Ly6C^lo^ monocytes was not reduced (Additional file 5: Figure S5). Since CCR2 is critical for the recruitment of circulating monocytes to the brain [8, 25, 34], we predicted that recruitment of Ly6C^hi^ monocytes would be reduced in CCR2^−/−^ mice at 3 days after MCAo, while Ly6C^lo^ Mo/MΦ would be present at 14 days only if the latter were recruited de novo from circulating CX3CR1^+^Ly6C^lo^ cells. In agreement with our findings in CX3CR1^GFP/+^CCR2^RFP/+^ mice, WT mice showed considerable infiltration of CD45^hi^CD11b^+^Ly6G^−^Ly6C^hi^ Mo/MΦ 3 days after MCAo (26 ± 5 × 10^3^ cell/hemisphere), while CCR2^−/−^ mice largely lacked infiltrating monocytes (0.10 ± 0.08 × 10^3^ cell/hemisphere) (Fig. 5a). Because the infarct volume, evaluated 3 days after MCAo, was slightly higher in CCR2^−/−^ (57 ± 4 mm^3^) than in WT mice (47 ± 2 mm^3^; *p* = 0.049, *n* = 6/group), the lack of monocyte recruitment to the brain was unlikely to be due to a reduced post-ischemic inflammatory response in CCR2^−/−^ mice. Fourteen days after MCAo, there was a large increase of Ly6C^lo^ Mo/MΦ in WT mice (25 ± 12 × 10^3^ cell/hemisphere), while only few Ly6C^lo^ Mo/MΦ were observed in the brains of CCR2^−/−^ mice (1.6 ± 0.3 × 10^3^ cell/hemisphere) (Fig. 5b). At this point, the tissue damage measured by brain atrophy tended to be higher in CCR2^−/−^ (13.2 ± 3.2 mm^3^) mice as compared to WT mice (7.7 ± 3.5 mm^3^, *p* = 0.2, *n* = 5–6/group) (Additional file 5: Figure S5).

**Fig. 6:**
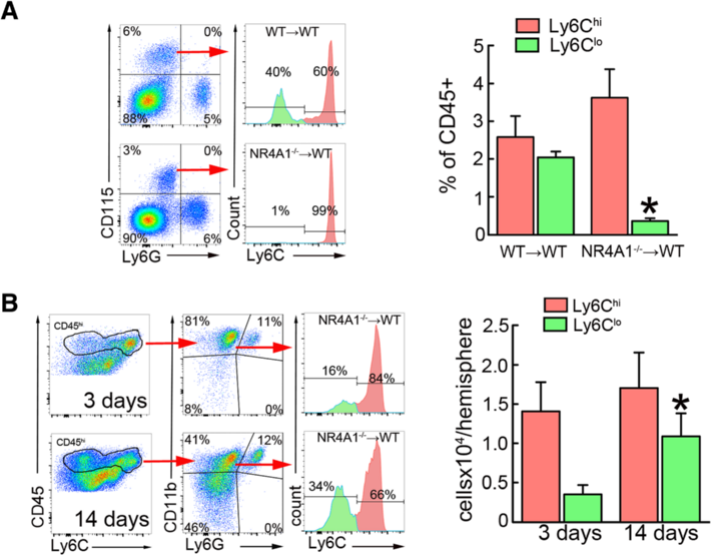
Brain Ly6C^lo^ Mo/MΦ develop independently of circulating Ly6C^lo^ monocytes after MCAo. **a** Blood flow cytometry analysis shows absence of Ly6C^lo^ monocytes, identified by expression of CD115 and low Ly6G expression, in NR4A1^−/−^ BM chimeric mice (*n* = 6 mice/group). *p < 0.05 vs. irradiated WT mice (WT → WT). **b** Brain flow cytometry analysis from NR4A1^−/−^ chimeric mice shows an increase of infiltrating Ly6C^lo^ Mo/MΦ, identified by high expression of CD45 and CD11b and low expression of Ly6G (CD45^hi^CD11b^+^Ly6G^**−**^), 14 days after MCAo compared to 3 days after MCAo. *p < 0.05 vs. 3 days

These results suggest that either Ly6C^lo^ Mo/MΦ derived from infiltrating CCR2^+^Ly6C^hi^ monocytes or that parenchymal CCR2^+^Ly6C^hi^ Mo/MΦ are required for the infiltration of CX3CR1^+^Ly6C^lo^ monocytes to occur. To address this issue, we studied Mo/MΦ kinetics in mice deficient for the orphan nuclear receptor NR4A1 (Nur77). NR4A1 is essential for the generation of Ly6C^lo^ monocytes and NR4A1^−/−^ mice lack circulating Ly6C^lo^ monocytes [16]. Since NR4A1 has been implicated in neuronal function [35], we used NR4A1^−/−^ chimeras lacking NR4A1 in BM-derived cells to avoid confounding effects of deletion of NR4A1 in neurons. As expected, Ly6C^lo^ monocytes were absent in the blood of NR4A1^−/−^ BM chimeric mice, while circulating Ly6C^hi^ monocytes were comparable in number to those of mice transplanted with WT BM (Fig. 6a). However, the number of brain Ly6C^lo^ Mo/MΦ significantly increased in NR4A1^−/−^ BM chimeric mice 14 days after MCAo compared to the number of Ly6C^lo^ Mo/MΦ at 3 days post-MCAo (Fig. 6b), consistent with the hypothesis that infiltrating CCR2^+^Ly6C^hi^ cells transdifferentiate into CX3CR1^+^Ly6C^lo^ Mo/MΦ at later times.

## Discussion

We have demonstrated that two main subsets of blood-derived macrophages accumulate in the post-ischemic brain within distinct temporal and spatial patterns. While CCR2^+^ Mo/MΦ spread through the ischemic lesion in the absence of CX3CR1^+^ Mo/MΦ at the early phase (3–5 days post-MCAo), the latter accumulated in the peri-infarct zone, encircling the lesion filled of CCR2^+^ Mo/MΦ at the late phase (14–28 days post-MCAo). Using mice lacking either CCR2^+^Ly6C^hi^ or CX3CR1^+^Ly6C^lo^ monocytes, we also provided evidence that CCR2^+^Ly6C^hi^ Mo/MΦ give rise to CX3CR1^+^Ly6C^lo^ Mo/MΦ independently of CX3CR1+ circulating monocytes. CX3CR1^+^Ly6C^lo^ Mo/MΦ can adopt three different myeloid phenotypes in the ischemic brain depending on their localization: ramified phenotype reminiscent of an intermediate state of activated microglia in the peri-infarct, amoeboid phenotype consistent with phagocytic macrophages in the infarct region, and elongated perivascular macrophages associated with blood vessels.

### Blood-derived macrophages accumulate sequentially in the ischemic brain

The number of both CX3CR1^GFP/+^ and CCR2^RFP/+^ Mo/MΦ on days 1 and 2 after ischemia was low and similar to the number in brains of sham animals. These data indicate that sizable infiltration of macrophages in the brain does not occur before several days after the ischemic event, as previously described [4, 36, 37]. While CCR2^+^ Mo/MΦ predominated during the first 3–5 days after stroke, CX3CR1^GFP/+^ Mo/MΦ number strikingly increased at 14 and 28 days. CCR2^+^ and CX3CR1^+^ Mo/MΦ accumulated in the ischemic brain with a distinctive biphasic profile. At day 3, we observed a drastic increase of CCR2^+^ Mo/MΦ, whereas CX3CR1^+^ Mo/MΦ only moderately increased. Nearly all CCR2^+^ Mo/MΦ were located in the infarct area, consistent with an active involvement in the inflammatory processes occurring in the territory most affected by ischemia. In contrast, the CX3CR1^+^ Mo/MΦ were mostly found at the infarct’s periphery, both rostrally and caudally. By 7 days, CX3CR1^+^ Mo/MΦ outnumbered CCR2^+^ Mo/MΦ in the infarct’s periphery and continued to increase reaching a maximum at 14 days. At 28 days, numbers of CX3CR1^+^ Mo/MΦ were still elevated, suggesting that Mo/MΦ participate in inflammatory processes also in the chronic phase of the injury.

Our data define two distinct regions within the chronic ischemic brain lesion: a cavitated core filled with CCR2^+^ and CX3CR1^+^ Mo/MΦ and a surrounding area of extensive neuronal loss heavily populated by CX3CR1^+^ but not CCR2^+^ Mo/MΦ.

### Phenotypic differentiation of CX3CR1^+^ Mo/MΦ occurs surrounding the ischemic lesion

While CCR2^+^ Mo/MΦ retained a round/amoeboid phenotype, commonly associated with phagocytic activity [38] throughout the time period studied, CX3CR1^+^ Mo/MΦ displayed three distinct phenotypes: amoeboid, ramified and elongated perivascular. Like CCR2^+^ Mo/MΦ, amoeboid CX3CR1^+^ Mo/MΦ were predominantly located in the center of the lesion. Interestingly, we observed an increased numbers of double-positive cells in this area, possibly indicating that the conversion of CCR2^+^ into CX3CR1^+^ Mo/MΦ occurs in the lesion center. The fact that we did not observe CCR2^+^ Mo/MΦ with any other morphology than amoeboid suggests that the morphological polarization of Mo/MΦ is linked to CCR2 down-regulation and concomitant up-regulation of CX3CR1. Indeed, in human monocytes, CCR2 is down-regulated as the monocytes differentiate into macrophages [39]. With the exception of the necrotic center, ramified CX3CR1^+^ Mo/MΦ were evenly distributed throughout the infarct without any apparent regional preference. Due to their high Iba1 expression and cytoplasmic arborization, these cells were essentially indistinguishable from activated microglia. Many of CX3CR1^+^ Mo/MΦ with elongated cell bodies were associated with cerebral blood vessels. These CX3CR1^+^ Mo/MΦ were strictly located on the abluminal side of the vessel and were also abluminal to pericytes and smooth muscle cells lining the vessel. Interestingly, CX3CR1^+^ Mo/MΦ did not cover all vessels uniformly but were associated with blood vessel that showed disruption of integrity as manifested by uneven or discontinuous smooth muscle cell coverage (Fig. 4c). While we can only speculate on their function, it is possible that vessel-associated CX3CR1^+^ Mo/MΦ support vascular integrity by physically strengthening the wall of vessels damaged by the ischemic injury. The recent findings that monocyte depleted mice showed a higher rate of hemorrhagic transformation after ischemic stroke support this view [8]. On the other hand, vessel-associated Mo/MΦ may participate in the removal of damaged vessels to allow vascular replacement by angiogenesis. While this possibility cannot be excluded, we did not see any intracellular inclusions positive for vascular markers (e.g. GLUT1, α-actin) that would indicate a phagocytic activity of these cells.

### Infiltration of inflammatory CCR2^+^Ly6C^hi^ may give rise to CX3CR1^+^Ly6C^lo^ in the ischemic brain

Several perivascular CX3CR1^+^Ly6C^lo^ Mo/MΦ were immunopositive for Ki67 3 days after MCAo but only few proliferative CX3CR1^+^Ly6C^lo^ Mo/MΦ, exhibiting mostly a ramified phenotype, were observed at later periods. Low rates of Mo/MΦ proliferation have also been reported in a mouse model of permanent cerebral ischemia [19]. Considering these low proliferation rates, it is more likely that the increased numbers of brain Mo/MΦ are the result of continuous recruitment of hematogenous monocytes than from cell proliferation in situ. Kokovay et al. [40] also reported that recruited bone marrow-derived cells into the brain expressing microglia markers were only occasionally proliferative. Interestingly, this study described a high number of Ki67^+^ cells associated with blood vessels 3–14 days after ischemia and between 10 and 20 % of the Ki67^+^ cells were identified as BM-derived pericytes. Contrary to the Kokovay study, however, we found that these perivascular cells expressed markers of the myeloid lineage but not pericytes, a discrepancy possibly due to the use of different pericyte markers (PDGFRβ in our study vs. desmin).

On the other hand, the increase in CX3CR1^GFP/+^CCR2^RFP/+^ double-positive cells in the ischemic lesion before the rise of CX3CR1^GFP/+^ single positive cells suggests that CX3CR1^+^Ly6C^lo^ derive from infiltrating CCR2^+^Ly6C^hi^ Mo/MΦ and not from a hematogenous source. This premise is supported by the observation that mice deficient in CCR2 did not exhibit significant numbers of both Ly6C^hi^ and Ly6C^lo^ Mo/MΦ in the ischemic brain, despite a normal number of circulating Ly6C^lo^ monocytes. Furthermore, in chimeric mice lacking the transcription factor NR4A1 in BM-derived cells, Ly6C^lo^ monocytes were drastically reduced in the circulation, but Ly6C^lo^ Mo/MΦ were still observed in the brain 14 days after stroke. Therefore, the Ly6C^lo^ Mo/MΦ present in the post-ischemic brain could not have originated from circulating Ly6C^lo^ monocytes. These observations raise the possibility that CX3CR1^+^Ly6C^lo^ Mo/MΦ differentiate from CCR2^+^Ly6C^hi^ Mo/MΦ in situ, rather than originate from infiltrating CX3CR1^+^Ly6C^lo^ blood monocytes. Our data are consistent with the observations by Gliem et al. [8] of reduced accumulation of differentiated macrophages (identified as Ly6c^lo^F4/80^hi^) in CCR2^−/−^ mice 6 days after ischemia. Thus, CCR2^+^Ly6C^hi^ Mo/MΦ could be precursors for CX3CR1^+^Ly6C^lo^ Mo/MΦ from which they develop through environmental cues provided by the ischemic brain tissue, as has been recently suggested [19]. The signals and their target receptors responsible for this phenotypic shift remain to be determined.

Because CX3CR1^+^Ly6C^lo^ Mo/MΦ have been linked to post-ischemic repair in several organs including the brain [8, 41–43], our findings have important implications for therapies targeting the migration of inflammatory leukocytes into the ischemic brain. Indeed, depletion of circulating CCR2 monocytes impairs long-term spontaneous behavioral recovery after stroke [37], while Ly6C^lo^ monocyte deficiency does not affect functional recovery and severity of injury after hypoxia/ischemia in mice [18]. All these findings suggest that inhibiting the recruitment of presumably proinflammatory CCR2^+^Ly6C^hi^ monocytes would also prevent the development of potentially protective CX3CR1^+^Ly6C^lo^ Mo/MΦ, which might affect repair processes during the subacute and chronic phase of ischemic brain injury. Therefore, therapies directed at interfering with monocyte recruitment into the ischemic brain must take into account the stage of evolution of the damage and the phenotype of the cells involved.

## Conclusions

In conclusion, we have investigated the time course and phenotypic characteristics of monocytes recruitment into the post-ischemic brain. We found that “inflammatory” monocytes (CCR2^+^Ly6C^hi^) are recruited into the brain in the acute phase of the damage resulting in early accumulation of CCR2^+^Ly6C^hi^ Mo/MΦ. A secondary accumulation of CX3CR1^+^Ly6C^lo^ Mo/MΦ ensues weeks later, which is most likely the result of a phenotypic switch of CCR2^+^Ly6C^hi^ Mo/MΦ rather than recruitment of blood borne “patrolling” monocytes (CX3CR1^+^Ly6C^lo^). The morphological diversity of CCR2^+^Ly6C^hi^ and CX3CR1^+^Ly6C^lo^ Mo/MΦ suggests a diversity of roles in the development and resolution of the damage. Although the molecular cues driving the phenotypic switch and its impact on tissue outcome remain to be defined, the data provide new insight into the complexities of monocyte trafficking into the brain with significant implications for immune-based approaches to ameliorate ischemic brain injury by modulating the cellular bases of post-ischemic inflammation.

## Additional files

Additional file 1: Figure S1. CX3CR1^GFP/+^ and CCR2^RFP/+^ infiltrating monocytes after stroke in head-shielded CCR2^RFP/+^CX3CR1^GFP/+^ chimeric mice. (A) Coronal brain section images of BM CCR2^RFP/+^CX3CR1^GFP/+^ chimeric mice 3 and 14 days after MCAo showing anatomical localization of accumulated CCR2^RFP/+^ (red) and CX3CR1^GFP/+^ (green) cells in the ischemic hemisphere. (B) Cell number quantification of infiltrating CX3CR1^GFP/+^ and CCR2^RFP/+^ in the ischemic hemisphere 3 and 14 days after MCAo. (C) Representative images and orthogonal views of co-staining of CX3CR1^GFP/+^ Mo/MΦ (green) with the proliferation marker Ki67 (red) showing CX3CR1^GFP/+^ Mo/MΦ 14 days after MCAo. (D) CX3CR1^GFP/+^ cells (green) show three different phenotypes: cells with an amoeboid shape (left), perivascular cells with elongated shape located along the vessels (center panels; transverse and longitudinal vessel cross sections, respectively) and cells with ramified processes (right) 14 days after MCAo. Blood vessels were immunostained with the endothelial marker GLUT1 (red). (PDF 4803 kb)
Additional file 2: Figure S2. CX3CR1^GFP/+^ Mo/MΦ do not differentiate into neurons, astrocytes, or oligodendrocytes. Staining for NeuN (A), GFAP (B), and Olig2 (C) shows that infiltrating CX3CR1^GFP/+^ cells in the peri-infarct or core of the ischemic hemisphere do not co-localize with neuronal, astrocytic, or oligodendrocyte markers 14 days after MCAo, respectively. Additionally, while CX3CR1^GFP/+^ cells, oligodendrocytes, and astrocytes vastly populate the ischemic core neurons were only present in the peri-infarct (A–C, right below panels). Scale bars represent 50 μm. (PDF 3837 kb)
Additional file 3: Figure S3. Infiltrating CX3CR1^GFP/+^ and CCR2^RFP/+^ cells are monocyte/macrophages. (A) Representative fluorescent merged images and orthogonal views of CX3CR1^GFP/+^ (green) and Iba1^+^ (red) ramified (upper panels) and amoeboid cells (lower panels) showing co-localization of both markers. The images were taken from the ischemic hemisphere at 14 days post-MCAo. Scale bar represent 20 μm. (B) Representative fluorescent merged images and orthogonal views of co-localization for the markers CCR2^GFP/+^ (red) and Iba1^+^ (blue). The images were taken from the ischemic hemisphere at 3 days post-MCAo. Scale bar represent 20 μm. (PDF 10117 kb)
Additional file 4: Figure S4. CX3CR1^GFP/+^ and CCR2^RFP/+^ monocytes overlap with Ly6C^lo^ and Ly6C^hi^ monocytes, respectively. Flow chart showing sequential gating to identify leukocyte subsets expressing GFP and/or RFP in the blood of CCR2^RFP/+^CX3CR1^GFP/+^ chimeric mice. While Ly6G^hi^ neutrophils were GFP/RFP negative, we identified three different subpopulations in CD45^+^ cells that showed green or/and red fluorescence: a subset of cells expressing high level of GFP and low level of RFP (GFP^hi^RFP^lo^, 1), a subset of cells expressing intermediate level of GFP and high level of RFP (GFP^int^RFP^hi^, 2), and a subset of cells that only express RFP (GFP^lo^RFP^hi^, 3). GFP^hi^RFP^lo^ and GFP^int^RFP^hi^ subsets were identified as Ly6C^lo^ and Ly6C^hi^ monocytes (CD45^hi^CD11b^+^Ly6G^−^), respectively. Additionally, a subset of GFP^lo^RFP^hi^ cells were identified mainly as NK cells and to a lesser extent as CD8^+^ cells. (PDF 791 kb)
Additional file 5: Figure S5. Monocyte blood analysis and brain injury quantification in CCR2^−/−^ mice after MCAo. (A) Blood flow cytometry analysis showed decreased numbers of Ly6C^hi^ monocytes, but not of Ly6C^lo^ monocytes, in CCR2^−/−^ mice as compared to WT mice (*n* = 5 mice/group). **p* < 0.05 vs. WT mice. (B) Ischemic lesion and brain atrophy were evaluated using T2-weighted MRI in WT and CCR2^−/−^ mice. Injury volume was evaluated by measuring hyperintense areas on T2 at 3 days post-MCAo (red outline), and brain atrophy was evaluated on T2 images by subtracting the volume of intact tissue in the ipsilateral hemisphere (red outline) from that in the contralateral hemisphere (blue outline) [44] at 14 days post-MCAo. (PDF 1172 kb)

## Abbreviations

BM: Bone marrow
CBF: Cerebral blood flow
CCR2: C-C chemokine receptor type 2
CNS: Central nervous system
CX3CR1: C-X(3)-C motif chemokine receptor
EGFP: Enhanced green fluorescent protein
FBS: Fetal bovine serum
FSC: Forward scatter profile
GFAP: Astrocytic marker glial fibrillary acidic protein
GFP: Green fluorescent protein
GLUT-1: Glucose transporter 1
Iba1: Lonized calcium binding adaptor molecule 1
Ly6C: Lymphocyte antigen 6 complex locus C1
MCA: Middle cerebral artery
MCAo: Middle cerebral artery occlusion
Mo/MΦ: Monocytes/macrophages
NDS: Normal donkey serum
PBS: Phosphate-buffered saline
PBST: PBS triton X-100
PCR: Polymerase chain reaction
PDGFRβ: Pericyte marker beta-type platelet-derived growth factor receptor
RFP: Red fluorescent protein
SSC: Side scatter profile
WT: Wild type
