# Supplementary figures and images for: Spatio-temporal profile, phenotypic diversity, and fate of recruited monocytes into the post-ischemic brain
Source: J Neuroinflammation. 2016 Nov 4;13:285. doi: 10.1186/s12974-016-0750-0 (PMC5097435; doi:10.1186/s12974-016-0750-0)

# Garcia-Bonilla et al. Supplemental Figure 1

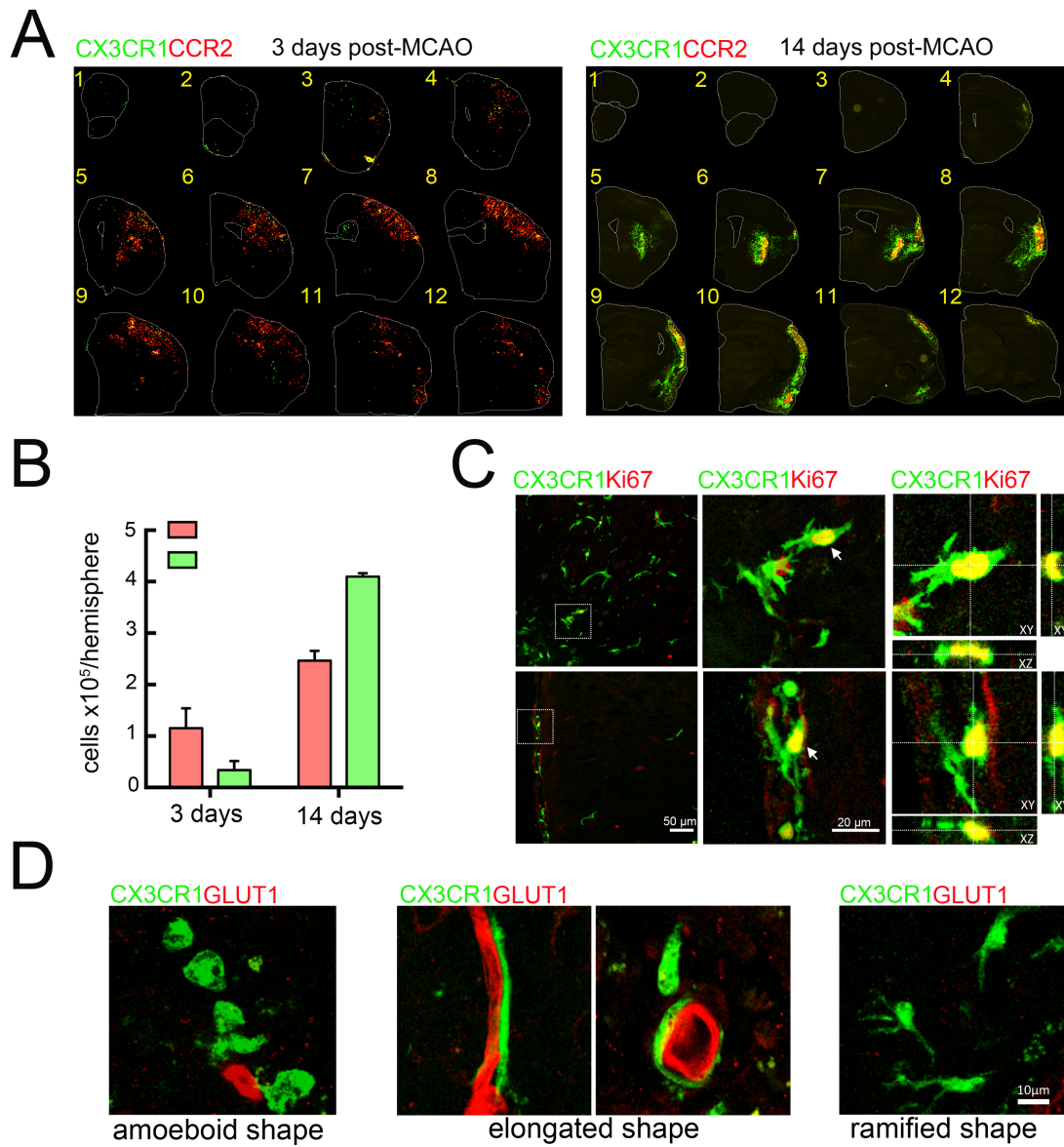

Supplement: Additional file 1: Figure S1. — CX3CR1GFP/+ and CCR2RFP/+ infiltrating monocytes after stroke in head-shielded CCR2RFP/+CX3CR1GFP/+ chimeric mice. (A) Coronal brain section images of BM CCR2RFP/+CX3CR1GFP/+ chimeric mice 3 and 14 days after MCAo showing anatomical localization of accumulated CCR2RFP/+ (red) and CX3CR1GFP/+ (green) cells in the ischemic hemisphere. (B) Cell number quantification of infiltrating CX3CR1GFP/+ and CCR2RFP/+ in the ischemic hemisphere 3 and 14 days after MCAo. (C) Representative images and orthogonal views of co-staining of CX3CR1GFP/+ Mo/MΦ (green) with the proliferation marker Ki67 (red) showing CX3CR1GFP/+ Mo/MΦ 14 days after MCAo. (D) CX3CR1GFP/+ cells (green) show three different phenotypes: cells with an amoeboid shape (left), perivascular cells with elongated shape located along the vessels (center panels; transverse and longitudinal vessel cross sections, respectively) and cells with ramified processes (right) 14 days after MCAo. Blood vessels were immunostained with the endothelial marker GLUT1 (red). (PDF 4803 kb) [file 12974_2016_750_MOESM1_ESM.pdf]

Garcia-Bonilla et al. Supplemental Figure 2

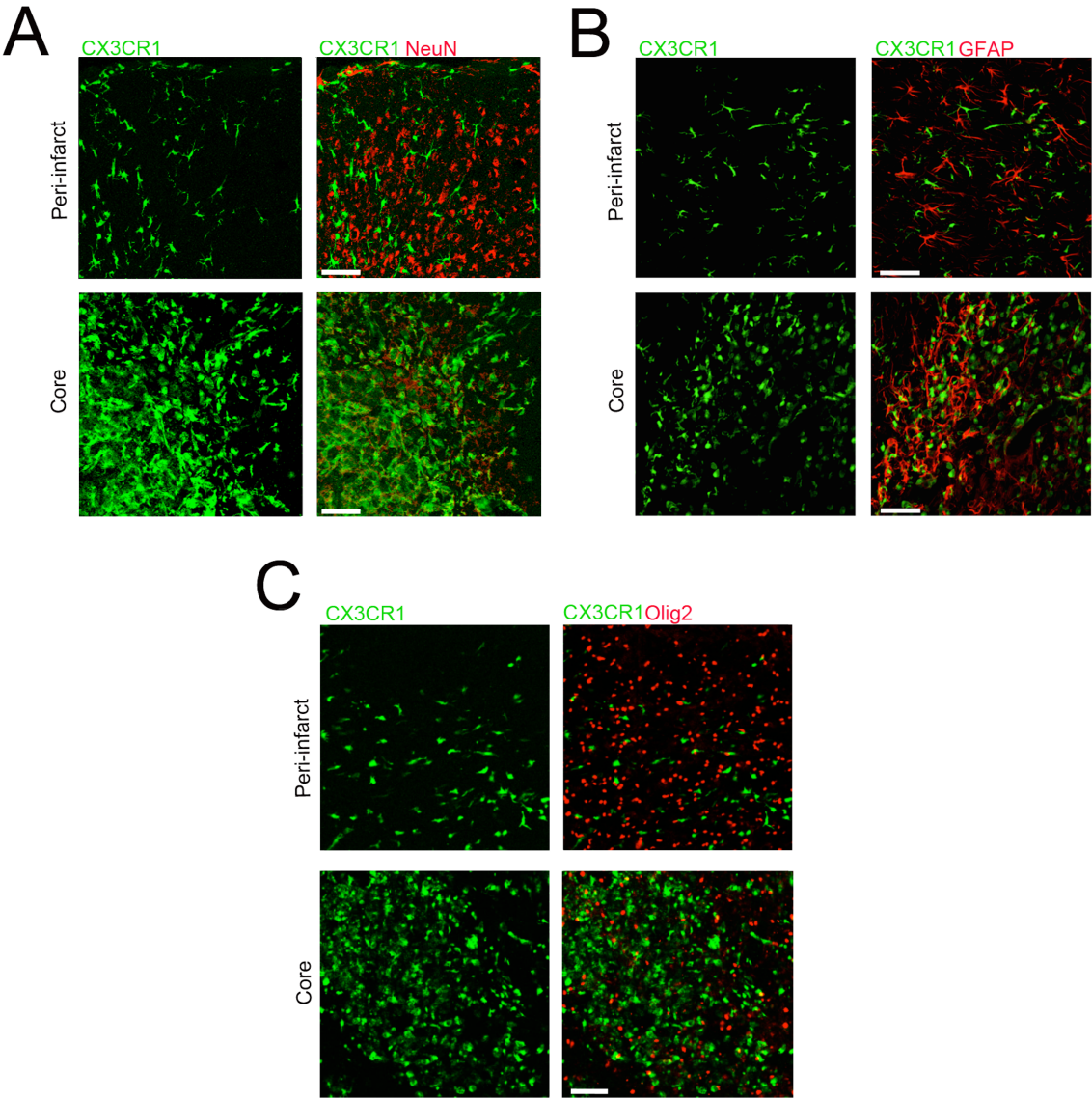

Supplement: Additional file 2: Figure S2. — CX3CR1GFP/+ Mo/MΦ do not differentiate into neurons, astrocytes, or oligodendrocytes. Staining for NeuN (A), GFAP (B), and Olig2 (C) shows that infiltrating CX3CR1GFP/+ cells in the peri-infarct or core of the ischemic hemisphere do not co-localize with neuronal, astrocytic, or oligodendrocyte markers 14 days after MCAo, respectively. Additionally, while CX3CR1GFP/+ cells, oligodendrocytes, and astrocytes vastly populate the ischemic core neurons were only present in the peri-infarct (A–C, right below panels). Scale bars represent 50 μm. (PDF 3837 kb) [file 12974_2016_750_MOESM2_ESM.pdf]

# Garcia-Bonilla et al. Supplemental Figure 3

A CX3CR1Iba1

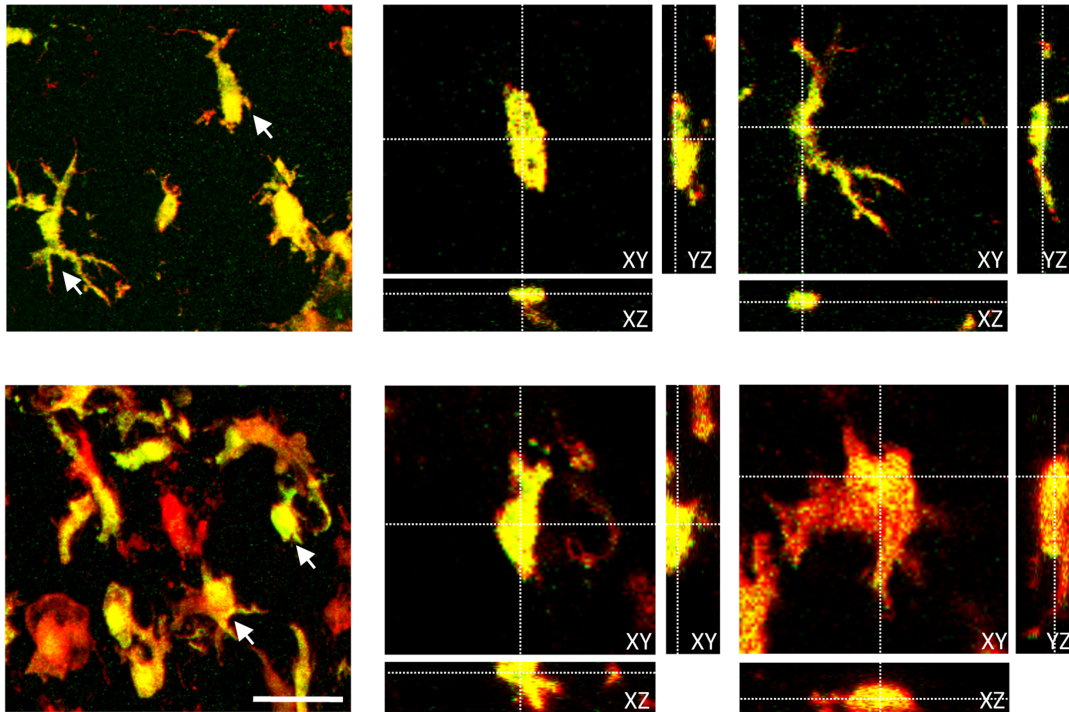

B CCR2Iba1

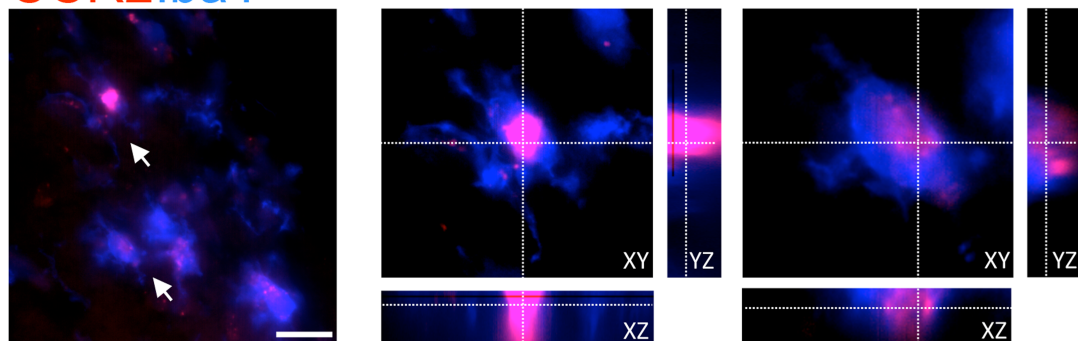

Supplement: Additional file 3: Figure S3. — Infiltrating CX3CR1GFP/+ and CCR2RFP/+ cells are monocyte/macrophages. (A) Representative fluorescent merged images and orthogonal views of CX3CR1GFP/+ (green) and Iba1+ (red) ramified (upper panels) and amoeboid cells (lower panels) showing co-localization of both markers. The images were taken from the ischemic hemisphere at 14 days post-MCAo. Scale bar represent 20 μm. (B) Representative fluorescent merged images and orthogonal views of co-localization for the markers CCR2GFP/+ (red) and Iba1+ (blue). The images were taken from the ischemic hemisphere at 3 days post-MCAo. Scale bar represent 20 μm. (PDF 10117 kb) [file 12974_2016_750_MOESM3_ESM.pdf]

Garcia-Bonilla et al. Supplemental Fig. 4

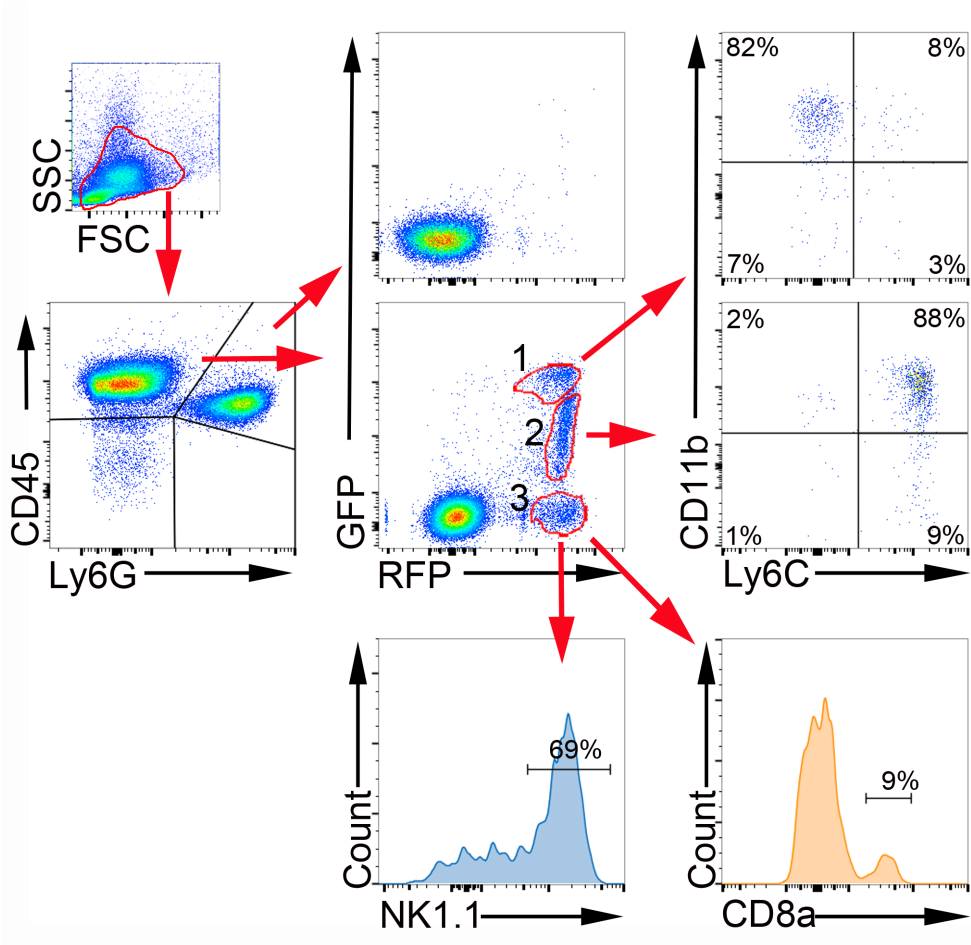

Supplement: Additional file 4: Figure S4. — CX3CR1GFP/+ and CCR2RFP/+ monocytes overlap with Ly6Clo and Ly6Chi monocytes, respectively. Flow chart showing sequential gating to identify leukocyte subsets expressing GFP and/or RFP in the blood of CCR2RFP/+CX3CR1GFP/+ chimeric mice. While Ly6Ghi neutrophils were GFP/RFP negative, we identified three different subpopulations in CD45+ cells that showed green or/and red fluorescence: a subset of cells expressing high level of GFP and low level of RFP (GFPhiRFPlo, 1), a subset of cells expressing intermediate level of GFP and high level of RFP (GFPintRFPhi, 2), and a subset of cells that only express RFP (GFPloRFPhi, 3). GFPhiRFPlo and GFPintRFPhi subsets were identified as Ly6Clo and Ly6Chi monocytes (CD45hiCD11b+Ly6G−), respectively. Additionally, a subset of GFPloRFPhi cells were identified mainly as NK cells and to a lesser extent as CD8+ cells. (PDF 791 kb) [file 12974_2016_750_MOESM4_ESM.pdf]

# Garcia-Bonilla et al. Supplemental Figure 5

**A**

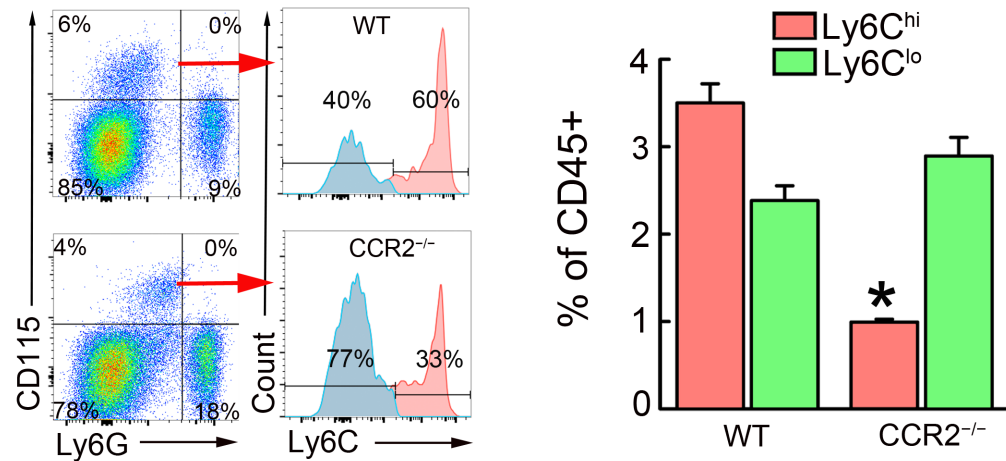

**B**

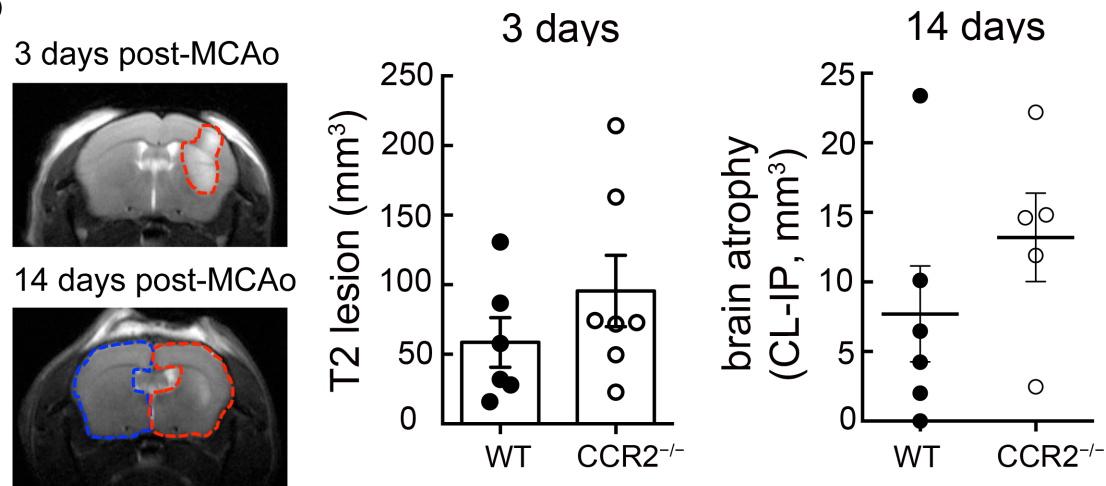

Supplement: Additional file 5: Figure S5. — Monocyte blood analysis and brain injury quantification in CCR2−/− mice after MCAo. (A) Blood flow cytometry analysis showed decreased numbers of Ly6Chi monocytes, but not of Ly6Clo monocytes, in CCR2−/− mice as compared to WT mice (n = 5 mice/group). *p < 0.05 vs. WT mice. (B) Ischemic lesion and brain atrophy were evaluated using T2-weighted MRI in WT and CCR2−/− mice. Injury volume was evaluated by measuring hyperintense areas on T2 at 3 days post-MCAo (red outline), and brain atrophy was evaluated on T2 images by subtracting the volume of intact tissue in the ipsilateral hemisphere (red outline) from that in the contralateral hemisphere (blue outline) [44] at 14 days post-MCAo. (PDF 1172 kb) [file 12974_2016_750_MOESM5_ESM.pdf]
